# Comparing 11 nutrition-inflammation indices for perioperative management and prognostic evaluation in non-small cell lung cancer patients

**DOI:** 10.3389/fnut.2025.1577563

**Published:** 2025-06-04

**Authors:** Yuanfang Wang, Hui Zhang, Fang Li, Zhao Zhang, Jing Wang, Qiuge Wu

**Affiliations:** Department of Pulmonary and Critical Care Medicine, The First Affiliated Hospital of Zhengzhou University, Zhengzhou, China

**Keywords:** non-small cell lung cancer, nutrition, inflammation, surgery, perioperative management, survival

## Abstract

**Background:**

Despite the establishment of multiple nutrition-inflammation indices, their performances in guiding clinical decision-making have not been systematically compared in patients with non-small cell lung cancer (NSCLC). This study aimed to identify the best nutrition-inflammation index for facilitating perioperative management and prognosis analysis in NSCLC patients.

**Methods:**

This study included NSCLC patients who underwent video-assisted thoracoscopic lobectomy as their primary treatment. Nutrition-inflammation indices were calculated based on blood tests and anthropometric measurements conducted within one week prior to surgery. A total of 11 nutrition-inflammation indices were compared for their performance in predicting perioperative and survival outcomes.

**Results:**

The cohort consisted of 805 patients, with a mean age of 60.3 years, including 388 females (48.2%) and 417 males (51.8%). Postoperative complications occurred in 152 patients (18.9%). The median follow-up time after surgery was 64.5 months. Most nutrition-inflammation indices demonstrated predictive values for perioperative complications, delayed hospital discharge, and survival outcomes, but with relatively low predictive accuracy. After adjusting for clinicopathological characteristics, most indices were no longer associated with these therapeutic outcomes. Among these indexes, the lymphocyte-to-monocyte ratio showed the best performance in predicting perioperative complications and delayed hospital discharge, while the geriatric nutritional risk index showed the best performance in predicting overall survival and disease-free survival.

**Conclusion:**

The current nutrition-inflammation indices demonstrated predictive values for therapeutic outcomes in NSCLC patients, but their utility in clinical practice may be limited due to generally weak independent associations. Future studies should focus on exploring more comprehensive nutrition-inflammation biomarkers for assisting clinical decision-making.

## Introduction

Systemic nutrition and inflammation indices have garnered significant attention in recent years due to their potential to guide treatments in patients with solid cancers (1, 2). These indices, derived from routine blood tests, reflect the complex interplay between nutrition, inflammation, and tumor progression (3, 4). They can be broadly classified into two groups: biochemical indices and biochemical-anthropometric indices. The biochemical indices include the albumin-to-globulin ratio (AGR) (5), lymphocyte-to-monocyte ratio (LMR) (6), and prognostic nutritional index (PNI) (7), while the biochemical-anthropometric indices include the advanced lung cancer inflammation index (ALI) (2) and the geriatric nutritional risk index (GNRI) (8). Although these indices are generally established based on retrospective cohort studies and have been investigated for their feasibility in predicting therapeutic outcomes in cancer patients, their results remain heterogeneous.

Lung cancer remains the most prevalent cancer and the leading cause of cancer-related mortality worldwide, with 2.48 million new cases and 1.82 million deaths reported in 2022 (9). Anatomical surgery, particularly video-assisted thoracoscopic surgery (VATS) lobectomy, is a cornerstone in the treatment of non-small cell lung cancer (NSCLC) (10, 11). Advances in surgical techniques and perioperative management have improved outcomes for many patients. However, the incidence of complications following VATS lobectomy remains significant (11, 12). Recurrence and metastasis still serve as the most common reasons for death and treatment failure in NSCLC patients undergoing VATS lobectomy (13). Preoperative nutritional and inflammatory status has been demonstrated to influence cancer response in NSCLC patients undergoing chemotherapy and immunotherapy (14, 15). Identifying risk factors and implementing effective interventions based on nutrition-inflammation status could be valuable for improving therapeutic outcomes in NSCLC patients undergoing VATS lobectomy.

Despite the establishment of multiple nutrition-inflammation indices, their performances in guiding perioperative management and prognosis assessment have not been systematically compared in NSCLC patients undergoing VATS lobectomy. Clinicians require clear indications regarding which index, with the optimal performance, should be adopted for predicting perioperative complications and recovery and long-term survival in NSCLC patients. Notably, these nutrition-inflammation indices may have differential effectiveness in predicting surgical outcomes and survival endpoints.

This study compared the performance of 11 nutrition-inflammation indices for guiding perioperative management and prognosis assessment in patients with NSCLC undergoing VATS lobectomy. The goal is to identify the best index for facilitating more effective perioperative management and improving long-term patient outcomes.

## Methods and materials

### Study design

This retrospective cohort study was conducted at the First Affiliated Hospital of Zhengzhou University. We extracted data from the hospital’s clinical database, focusing on NSCLC patients undergoing VATS lobectomy between January 2016 and December 2018. Data collection was prospective, while analysis was performed retrospectively. Preoperative nutrition and inflammation parameters were assessed based on blood tests and anthropometric measurements conducted within one week prior to surgery. The study protocol was approved by the Ethics Committee Board of the First Affiliated Hospital of Zhengzhou University (approval no. 2024-KY-1756-001). Informed consent had been obtained from all patients for the use of their data in institutional databases. The study adhered to the guidelines of the Strengthening the Reporting of Observational Studies in Epidemiology (STROBE) Statement (16).

### Participants

Patients with NSCLC, aged 18 years or older, who underwent anatomic VATS lobectomy were consecutively enrolled in both the development and validation cohorts. The exclusion criteria were as follows: (1) patients who underwent bilobectomy or sleeve lobectomy; (2) those who received preoperative anticancer treatment; (3) those with non-radical resection; (4) those with active infection within two weeks before surgery; (5) those with a history of thoracic or abdominal surgery within the past year; (6) those with a history of cancer within the past five years; (7) those with liver or kidney dysfunction; (8) those with comorbidities involving the rheumatic, immune, hematologic, or lymphatic systems; and (9) those with incomplete data required for analysis.

### Treatment strategy

Standard preoperative evaluation included thoracic computed tomography (CT), cardiopulmonary function tests, abdominal and adrenal gland ultrasonography, brain magnetic resonance imaging (MRI), and bone scans. For patients with enlarged mediastinal lymph nodes, endobronchial ultrasound-guided transbronchial needle aspiration or mediastinoscopy biopsy was performed. Positron emission tomography/CT (PET/CT) was used to detect suspected metastasis. Cancer staging or restaging was based on the 8th edition of the American Joint Committee on Cancer (AJCC) tumor, node, and metastasis (TNM) classification system. The standard surgical approach was anatomic VATS lobectomy with hilar and mediastinal lymph node dissection, performed by experienced thoracic surgeons. Postoperative care included management of fluid and electrolyte balance, nutritional support, pulmonary exercises, and physical rehabilitation.

### Nutrition-inflammation indexes

Systemic nutrition and inflammation parameters were evaluated from routine blood tests conducted within one week before surgery. These parameters included total protein, serum albumin, serum globulin, total cholesterol, hemoglobin, total neutrophils, total lymphocytes, total monocytes, and total platelets in peripheral blood. Eleven nutrition-inflammation indexes were calculated using these biochemical parameters, either alone or in combination with anthropometric parameters (detailed in Supplementary Table 1). The biochemical indexes included the AGR (5), LMR (6), PNI (7), controlling nutritional status score (COUNT) (17), neutrophil-to-lymphocyte ratio (NLR) (18), neutrophil-to-platelet ratio (NPR) (19), platelet-to-lymphocyte ratio (PLR) (20), systemic immune-inflammation index (SII) (21), and systemic inflammation response index (SIRI) (22). The biochemical-anthropometric indexes included the ALI (2) and GNRI (8). To investigate the changes in nutrition-inflammation indicators surrounding surgery, medical records within 4 to 6 weeks after surgery were analyzed and compared to those before surgery.

### Endpoints

The study aimed to evaluate the efficacy of nutrition-inflammation indexes in guiding perioperative management and predicting survival outcomes in NSCLC patients. For perioperative management, the primary endpoint was the predictive accuracy of these indexes for overall complications, while the secondary endpoint was their predictive accuracy for delayed hospital discharge. Postoperative complications were defined according to the Common Terminology Criteria for Adverse Events (CTCAE) version 5.0 (23). Discharge criteria included stable vital signs, ability to take oral feeds, absence of complications requiring hospital treatment, unassisted ambulation, and manageable pain with oral analgesics. Delayed discharge was defined as a postoperative hospital stay exceeding the upper tertile of the cohort’s distribution. Patients were closely followed up within four weeks post-discharge to detect late complications or other issues.

For survival analysis, the primary endpoint was the predictive accuracy of nutrition-inflammation indexes for overall survival (OS) following VATS lobectomy. Secondary endpoints included their predictive accuracy for disease-free survival (DFS) and cancer-specific survival (CSS). OS was calculated from the time of surgery to death from any cause. DFS was calculated from the time of surgery to the first recurrence of the index cancer or death from any cause. CSS was calculated from the time of surgery to death specifically caused by lung cancer.

### Statistical analysis

Categorical data were presented as frequencies (percentages), while continuous data were expressed as means (standard deviations, SDs) or medians (interquartile ranges, IQRs). Group differences were evaluated using ANOVA, Pearson’s chi-squared tests, Fisher’s exact tests, Mann–Whitney U tests, or Kruskal–Wallis tests, as appropriate. The changes in nutrition-inflammation indicators following surgery were evaluated using the paired sample Wilcoxon signed-rank test. Receiver operating characteristic (ROC) curves were used to assess the predictive accuracy of nutrition-inflammation indexes for the endpoints, with the area under the curve (AUC) indicating performance. Survival analysis was conducted using the Kaplan–Meier method, with log-rank statistics employed for comparison. Multivariable analysis was conducted using logistic regression and Cox proportional hazards regression models. Odds ratios (ORs) and hazard ratios (HRs) with 95% confidence intervals (CIs) were reported. A two-sided *p*-value of less than 0.05 was considered statistically significant. All analyses were performed using IBM SPSS Statistics for Windows (version 22.0, IBM Corp., Armonk, NY, United States) and the R programming environment (version 4.2.0, R Core Team, Vienna, Austria).

## Results

### Patients’ characteristics

**Figure 1:**
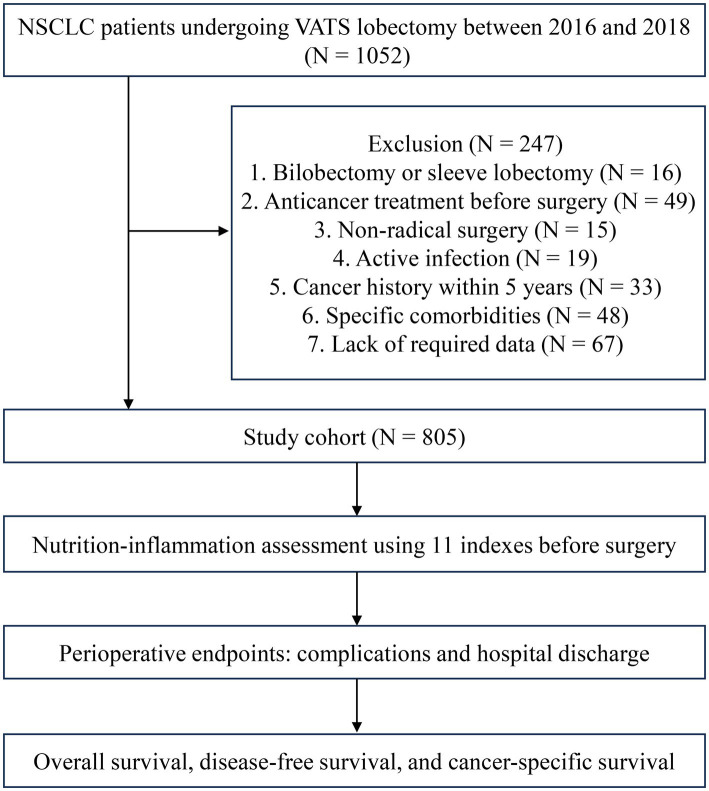
Study design and patient recruitment flowchart. This flowchart illustrates the process of the study design and the recruitment of patients for both development and validation cohorts. NSCLC: non-small cell lung cancer; VATS: the video-assisted thoracoscopic surgery.

During the study period, a total of 1,052 patients underwent VATS lobectomy at the institute and were assessed for inclusion. Of these, 247 patients were excluded for specified reasons. The remaining 805 patients constituted the study cohort (Figure 1). Detailed clinicopathological data of the patients are presented in Table 1. All patients underwent radical VATS lobectomy as the primary treatment. Postoperative complications occurred in 152 patients (18.9%), with a median chest tube duration of 5 days (IQR: 3–8) and a median postoperative hospital stay of 7 days (IQR: 6–10). The median follow-up time after surgery was 64.5 months (IQR: 30.2–79.8).

**Table 1:** Characteristics of the included patients.

Characteristics	Total (*N* = 805)
Demographic data	
Age, years	60.3 ± 11.4
Gender (female)	388 (48.2)
Smoking history	323 (40.1)
Charlson comorbidity index ≥ 3	137 (17.0)
Comorbidities	
Cardiovascular disease	108 (13.4)
Chronic obstructive pulmonary disease	51 (6.3)
Diabetes	90 (11.2)
FEV1, % predicted values	90.8 (75.6–105.0)
FEV1/FVC, %	78.5 (72.2–82.0)
Body mass index (BMI), kg/m^2^	24.2 (22.1–26.0)
Underweight (BMI < 20)	73 (9.1)
Normal weight (BMI: 20–25)	420 (52.2)
Overweight/Obesity (BMI ≥ 25)	312 (38.6)
Nutrition/inflammation items	
Total protein, g/L	67.5 (64.0–71.9)
Serum albumin, g/L	42.0 (40.0–44.6)
Serum globulin, g/L	25.4 (22.9–28.2)
Total cholesterol, mmol/L	4.59 (4.05–5.26)
Hemoglobin, g/L	134 (126–145)
Total neutrophils, /mm^3^	3,420 (2720–4,220)
Total lymphocytes, /mm^3^	1720 (1410–2,155)
Total monocytes, /mm^3^	400 (330–510)
Total platelet, ×10^3^/mm^3^	201 (167–238)
Nutrition-inflammation indexes	
PNI	51.3 (48.2–54.5)
COUNT	1 (0–2)
AGR	1.67 (1.51–1.84)
NLR	1.91 (1.45–2.60)
PLR	113.6 (90.7–144.3)
NPR, ×10^−2^	1.70 (1.30–2.20)
LMR	4.30 (3.29–5.48)
SIRI	0.783 (0.530–1.138)
SII	379 (273–544)
GNRI	109 (103–113)
ALI	52.4 (39.0–71.2)
Surgical parameters	
Operative time, min	180 (150–210)
Estimated blood loss, ml	100 (50–150)
Cancer characteristics	
Tumor location: right/left	497/308 (61.7/38.3)
Histology: adenocarcinoma/SCC/others	604/164/37 (75.0/20.4/4.6)
Multiple primary cancer	68 (8.4)
Pleural invasion	260 (32.3)
Vascular invasion	78 (9.7)
Pathological TNM stage: IA/IB/II/III	303/230/131/141 (37.6/28.6/16.3/17.5)
Postoperative endpoints	
Overall complications	152 (18.9)
Chest tube duration, days	5.0 (3.0–8.0)
Postoperative hospital stay, days	7.0 (6.0–10.0)

Data are mean ± standard deviation, number (percentage), or median (interquartile range). AGR: albumin-to-globulin ratio; ALI: advanced lung cancer inflammation index; CONUT: controlling nutritional status score; FEV1: forced expiratory volume in 1 s; FVC: forced vital capacity; GNRI: geriatric nutritional risk index; LMR: lymphocyte-to-monocyte ratio; NLR: neutrophil-to-lymphocyte ratio; NPR: neutrophil-to-platelet ratio; PLR: platelet-to-lymphocyte ratio; PNI: prognostic nutritional index; SCC: squamous cell carcinoma; SII: systemic immune-inflammation index; SIRI: systemic inflammation response index.

### Nutrition-inflammation indexes and perioperative endpoints

**Figure 2:**
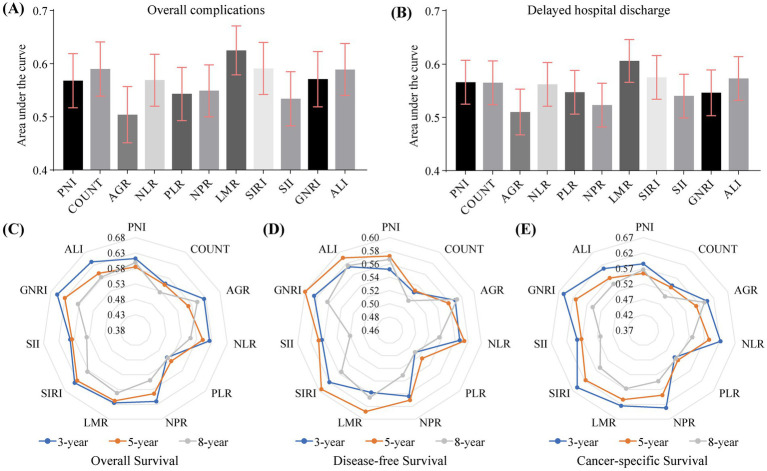
Predictive values of systemic nutrition-inflammation indicators for perioperative and survival outcomes in NSCLC patients. The detailed results are presented in Supplementary Table 2. **(A,B)** Areas under the curve (AUC) and 95% confidence intervals derived from the Receiver Operating Characteristic (ROC) curves were used to evaluate the predictive values of nutrition-inflammation indicators for the incidence of postoperative complications **(A)** and delayed hospital discharge **(B)**. **(C,E)** Time-dependent ROC curves were employed to assess the predictive values of nutrition-inflammation indicators for overall survival **(C)**, disease-free survival **(D)**, and cancer-specific survival **(E)**, AUC values at the specific time points of 3, 5, and 8 years post-surgery are presented in radar plots. AGR: albumin-to-globulin ratio; ALI: advanced lung cancer inflammation index; CONUT: controlling nutritional status score; GNRI: geriatric nutritional risk index; LMR: lymphocyte-to-monocyte ratio; NLR: neutrophil-to-lymphocyte ratio; NPR: neutrophil-to-platelet ratio; PLR: platelet-to-lymphocyte ratio; PNI: prognostic nutritional index; SII: systemic immune-inflammation index; SIRI: systemic inflammation response index.

Most nutrition-inflammation indexes demonstrated predictive values for the incidence of postoperative complications and delayed hospital discharge (Supplementary Table 2; Figure 2). However, the AUC values of these indexes were generally low. Notably, the LMR exhibited the optimal AUC values in predicting perioperative endpoints compared to other indexes (Figures 2A,B). Most indexes showed significant predictive associations with the incidence of postoperative complications and delayed hospital discharge in univariable analysis (Table 2). However, after adjusting for clinicopathological characteristics, particularly age and gender, most indexes were no longer associated with perioperative endpoints. Notably, both the COUNT score and LMR were independently associated with the incidence of postoperative complications, and only the LMR was independently associated with delayed hospital discharge.

**Table 2:** Prognostic values of clinicopathological characteristics for perioperative endpoints in NSCLC patients undergoing VATS lobectomy (*N* = 805).

		The incidence of overall complications^a^				Delayed hospital discharge^a^			
Characteristics	Comparisons	Univariable analysis		Multivariable analysis		Univariable analysis		Multivariable analysis	
		OR (95%CI)	*p* value	OR (95%CI)	*P* value	HR (95%CI)	*P* value	HR (95%CI)	*P* value
Demographic data									
Age	Per 10 years	1.92 (1.58–2.34)	<0.001	1.85 (1.52–2.25)	<0.001	1.53 (1.32–1.77)	<0.001	1.35 (1.15–1.59)	<0.001
Gender (female)	Female vs. male	0.46 (0.32–0.66)	<0.001	0.52 (0.35–0.76)	0.001	0.51 (0.39–0.69)	<0.001	0.62 (0.45–0.87)	0.005
Smoking history	Yes vs. no	1.81 (1.27–2.59)	<0.001	-	-	1.71 (1.27–2.29)	<0.001	-	-
Charlson comorbidity index	≥3 vs. <3	2.14 (1.41–3.28)	<0.001	-	-	1.59 (1.09–2.31)	0.016	-	-
Cardiovascular disease	Yes vs. no	1.81 (1.14–2.87)	0.013	-	-	1.22 (0.81–1.86)	0.35	-	-
COPD	Yes vs. no	1.51 (0.78–2.91)	0.22	-	-	1.72 (0.97–3.05)	0.062	-	-
Diabetes	Yes vs. no	0.92 (0.52–1.63)	0.77	-	-	1.03 (0.65–1.64)	0.89	-	-
FEV1, %	Per 1%	1.00 (0.99–1.01)	0.89	-	-	0.99 (0.99–1.00)	0.057	-	-
FEV1/FVC, %	Per 1%	1.00 (0.98–1.01)	0.37	-	-	0.98 (0.98–0.99)	0.001	0.99 (0.98–1.00)	0.044
Body mass index	Per 1 kg/m^2^	0.95 (0.89–1.00)	0.055	-	-	0.98 (0.93–1.02)	0.34	-	-
Surgical parameters									
Operative time	Per 10 min	1.00 (0.97–1.04)	0.84	-	-	1.03 (1.01–1.06)	0.013	-	-
Estimated blood loss	Per 10 mL	1.02 (1.01–1.03)	0.007	-	-	1.04 (1.03–1.05)	<0.001	1.04 (1.02–1.05)	<0.001
Cancer characteristics									
Tumor location	Left vs. right	1.14 (0.79–1.63)	0.49	-	-	1.22 (0.90–1.64)	0.20	-	-
Histology:	AC vs. others	0.57 (0.38–0.87)	0.009	-	-	0.63 (0.44–0.90)	0.011	-	-
Multiple primary cancer	Yes vs. no	0.91 (0.48–1.75)	0.91	-	-	0.71 (0.41–1.23)	0.22	-	-
Pleural invasion	Yes vs. no	0.85 (0.58–1.25)	0.41	-	-	0.91 (0.67–1.24)	0.55	-	-
Vascular invasion	Yes vs. no	0.68 (0.35–1.33)	0.26	-	-	0.67 (0.40–1.12)	0.13	-	-
Pathological TNM stage	IB vs. IA	0.95 (0.61–1.47)	0.82	-	-	1.28 (0.89–1.84)	0.18	-	-
	II vs. IA	0.88 (0.52–1.50)	0.64	-	-	1.19 (0.77–1.83)	0.44	-	-
	III vs. IA	0.98 (0.59–163)	0.94	-	-	1.36 (0.90–2.06)	0.15	-	-
The existing systemic nutrition/inflammation indicators^b^									
PNI	Per 1 unit	0.95 (0.91–0.99)	0.009	-	-	0.95 (0.92–0.98)	0.001	-	-
COUNT	Per 1 score	1.30 (1.12–1.50)	<0.001	1.18 (1.10–1.37)	0.038	1.20 (1.06–1.36)	0.004	-	-
AGR	Per 1 unit	1.05 (0.55–1.99)	0.89	-	-	1.15 (0.68–1.96)	0.60	-	-
NLR	Per 1 unit	1.17 (1.01–1.36)	0.039	-	-	1.15 (1.01–1.32)	0.040	-	-
PLR	Per 10 units	1.01 (0.98–1.05)	0.51	-	-	1.03 (1.00–1.06)	0.036	-	-
NPR	Per 0.01 units	1.12 (0.94–1.34)	0.21	-	-	1.03 (0.87–1.21)	0.76	-	-
LMR	Per 1 unit	0.74 (0.66–0.84)	<0.001	0.85 (0.75–0.97)	0.013	1.04 (1.02–1.06)	0.001	1.03 (1.00–1.06)	0.041
SIRI	Per 1 unit	1.44 (1.14–1.83)	0.003	-	-	1.37 (1.10–1.70)	0.005	-	-
SII	Per 50 units	1.01 (0.98–1.03)	0.60	-	-	1.02 (1.00–1.04)	0.10	-	-
GNRI	Per 1 unit	0.97 (0.95–0.99)	0.010	-	-	0.98 (0.96–1.00)	0.040	-	-
ALI	Per 10 units	0.88 (0.82–0.95)	0.001	-	-	0.91 (0.86–0.96)	0.001	-	-

a Logistic regression analyses were performed to evaluate the association between clinicopathological characteristics and perioperative endpoints. The outcomes are presented as odds ratios (ORs) with 95% confidence intervals (CIs). Variables with a *P*-value of less than 0.10 in the univariate analysis were selected for inclusion in the multivariate regression models, using the backward conditional methods.

b All systemic nutrition/inflammation indicators were individually incorporated into the multivariate analyses.

AGR: albumin-to-globulin ratio; ALI: advanced lung cancer inflammation index; CONUT: controlling nutritional status score; COPD: chronic obstructive pulmonary disease; FEV1: forced expiratory volume in 1 s; FVC: forced vital capacity; GNRI: geriatric nutritional risk index; LMR: lymphocyte-to-monocyte ratio; NLR: neutrophil-to-lymphocyte ratio; NPR: neutrophil-to-platelet ratio; PLR: platelet-to-lymphocyte ratio; PNI: prognostic nutritional index; SII: systemic immune-inflammation index; SIRI: systemic inflammation response index.

### Nutrition-inflammation indexes and survival outcomes

Most nutrition-inflammation indexes showed predictive values for OS, DFS, and CSS after VATS lobectomy (Supplementary Table 2; Figure 2). Focusing on the 8-year outcomes, the predictive values of these indexes for OS were generally better than those for DFS and CSS. However, the AUC values of these indexes were generally low, and the GNRI exhibited the optimal AUC values in predicting OS, DFS, and CSS profiles across different time points (Figures 2C–E). Most indexes showed significant predictive values for OS, DFS, and CSS in univariable analysis (Table 3), with the significance generally attenuating across OS, DFS, and CSS. After adjusting for clinicopathological characteristics, particularly age, vascular invasion, and pathological cancer stage, most indexes were no longer significantly associated with survival outcomes. Specifically, the NLR, NPR, LMR, SIRI, and GNRI were independently predictive of OS, the GNRI was independently predictive of DFS, the SIRI was non-significantly predictive of DFS, while no indexes were independently predictive of CSS.

**Table 3:** Prognostic values of clinicopathological characteristics for survival outcomes in NSCLC patients undergoing VATS lobectomy (*N* = 805).

		Overall survival^a^				Disease-free survival^a^				Cancer-specific survival^a^			
Parameters	Comparisons	Univariable analysis		Multivariable analysis		Univariable analysis		Multivariable analysis		Univariable analysis		Multivariable analysis	
		HR (95%CI)	*P* value	HR (95%CI)	*P* value	HR (95%CI)	*P* value	HR (95%CI)	*P* value	HR (95%CI)	*P* value	HR (95%CI)	*P* value
Demographic data													
Age	Per 10 years	1.56 (1.36–1.79)	<0.001	1.43 (1.24–1.65)	<0.001	1.46 (1.29–1.64)	<0.001	1.37 (1.21–1.55)	<0.001	1.44 (1.24–1.67)	<0.001	1.32 (1.12–1.55)	0.001
Gender (female)	Female vs. male	0.64 (0.49–0.85)	0.002	-	-	0.77 (0.61–0.98)	0.035	-	-	0.75 (0.55–1.01)	0.061	-	-
Smoking history	Yes vs. no	1.62 (1.24–2.12)	<0.001	-	-	1.46 (1.16–1.85)	0.002	-	-	1.44 (0.07–1.94)	0.017	-	-
Charlson comorbidity index	≥3 vs. <3	1.67 (1.22–2.27)	0.001	-	-	1.74 (1.33–2.29)	<0.001	-	-	1.58 (1.11–2.24)	0.011	-	-
Cardiovascular disease	Yes vs. no	1.15 (0.79–1.68)	0.46	-	-	1.22 (0.88–1.69)	0.23	-	-	1.05 (0.68–1.62)	0.81	-	-
COPD	Yes vs. no	1.34 (0.83–2.17)	0.24	-	-	1.50 (0.99–2.26)	0.053	-	-	1.60 (0.97–2.63)	0.067	-	-
Diabetes	Yes vs. no	1.21 (0.82–1.78)	0.35	-	-	1.09 (0.76–1.56)	0.65	-	-	1.13 (0.72–1.77)	0.59	-	-
FEV1, %	Per 1%	1.00 (0.98–1.01)	0.26	-	-	1.00 (1.00–1.01)	0.31	-	-	1.00 (1.00–1.01)	0.46	-	-
FEV1/FVC, %	Per 1%	0.99 (0.99–1.00)	0.16	-	-	1.00 (0.99–1.00)	0.13	-	-	0.99 (0.99–1.00)	0.14	-	-
Body mass index	Per 1 kg/m^2^	0.97 (0.92–1.01)	0.12	-	-	0.97 (0.93–1.00)	0.077	-	-	0.98 (0.94–1.03)	0.52	-	-
Surgical parameters													
Operative time	Per 10 min	1.06 (1.04–1.08)	<0.001	1.04 (1.01–1.06)	0.002	1.04 (1.02–1.06)	<0.001	-	-	1.05 (1.02–1.08)	<0.001	1.03 (1.01–1.06)	0.012
Blood loss	Per 10 ml	1.02 (1.01–1.03)	<0.001	-	-	1.02 (1.01–1.03)	<0.001	-	-	1.02 (1.01–1.03)	0.002	-	-
Cancer characteristics													
Tumor location	Left vs. right	1.01 (0.83–1.44)	0.52	-	-	1.02 (0.80–1.30)	0.90	-	-	1.03 (0.76–1.40)	0.86	-	-
Histology	AC vs. others	0.65 (0.48–0.88)	0.006	-	-	0.78 (0.59–1.04)	0.088	-	-	0.89 (0.61–1.29)	0.54	-	-
Multiple primary cancer	Yes vs. no	1.05 (0.65–1.70)	0.86	-	-	0.88 (0.56–1.39)	0.59	-	-	1.00 (0.58–1.74)	0.99	-	-
Pleural invasion	Yes vs. no	1.61 (1.22–2.11)	0.001	-	-	1.70 (1.34–2.16)	<0.001	1.28 (0.99–1.67)	0.063	1.81 (1.34–2.44)	<0.001	-	-
Vascular invasion	Yes vs. no	2.62 (1.84–3.72)	<0.001	1.48 (1.03–2.14)	0.034	2.68 (1.96–3.66)	<0.001	1.43 (1.02–1.99)	0.036	2.85 (1.95–4.17)	<0.001	1.49 (0.99–2.24)	0.058
Pathological TNM stage	IB vs. IA	2.25 (1.40–3.63)	0.001	2.02 (1.25–3.25)	0.004	2.32 (1.58–3.43)	<0.001	1.91 (1.26–2.91)	0.002	2.99 (1.68–5.30)	<0.001	2.75 (1.53–4.96)	0.001
	II vs. IA	6.75 (4.29–10.6)	<0.001	5.57 (3.52–8.80)	<0.001	5.23 (3.62–7.84)	<0.001	4.37 (2.92–6.54)	<0.001	8.60 (4.95–14.9)	<0.001	6.88 (3.86–12.3)	<0.001
	III vs. IA	9.24 (5.97–14.3)	<0.001	7.80 (4.98–12.2)	<0.001	8.50 (5.90–12.3)	<0.001	6.93 (4.70–10.2)	<0.001	12.5 (7.34–21.3)	<0.001	10.6 (6.04–18.5)	<0.001
Postoperative complications													
Overall complications	Yes vs. no	1.27 (0.92–1.74)	0.15	-	-	1.33 (1.00–1.76)	0.047	-	-	1.05 (0.72–1.53)	0.81	-	-
Postoperative hospital stay	Per 1 day	1.03 (1.00–1.05)	0.27	-	-	1.03 (1.01–1.05)	0.014	-	-	1.02 (0.99–1.05)	0.33	-	-
Immuno-nutritional indicators^b^													
PNI	Per 1 unit	0.95 (0.92–0.97)	<0.001	-	-	0.96 (0.94–0.99)	0.004	-	-	0.97 (0.94–1.00)	0.040	-	-
COUNT	Per 1 score	1.12 (1.01–1.25)	0.037	-	-	1.07 (0.97–1.18)	0.19	-	-	1.06 (0.93–1.20)	0.41	-	-
AGR	Per 1 unit	0.43 (0.26–0.72)	0.001	-	-	0.54 (0.34–0.85)	0.009	-	-	0.46 (0.29–0.82)	0.008	-	-
NLR	Per 1 unit	1.20 (1.09–1.32)	<0.001	1.12 (1.01–1.25)	0.039	1.14 (1.04–1.25)	0.006	-	-	1.14 (1.01–1.29)	0.029	-	-
PLR	Per 10 units	1.02 (0.99–1.04)	0.25	-	-	1.01 (0.99–1.03)	0.38	-	-	1.00 (0.97–1.03)	0.92	-	-
NPR	Per 0.01 units	1.14 (1.05–1.25)	0.003	1.13 (1.01–1.26)	0.032	1.10 (1.01–1.21)	0.039	-	-	1.13 (1.02–1.25)	0.017	-	-
LMR	Per 1 unit	0.84 (0.77–0.91)	<0.001	0.90 (0.83–0.98)	0.020	0.89 (0.83–0.96)	0.002	-	-	0.88 (0.80–0.96)	0.006	-	-
SIRI	Per 1 unit	1.48 (1.28–1.72)	<0.001	1.28 (1.09–1.50)	0.018	1.36 (1.18–1.58)	<0.001	1.15 (0.98–1.35)	0.080	1.39 (1.17–1.66)	<0.001	-	-
SII	Per 50 units	1.02 (1.00–1.04)	0.017	-	-	1.01 (1.00–1.03)	0.11	-	-	1.01 (0.99–1.04)	0.19	-	-
GNRI	Per 1 unit	0.97 (0.95–0.98)	<0.001	0.98 (0.96–0.99)	0.012	0.97 (0.96–0.99)	<0.001	0.98 (0.97–0.99)	0.029	0.98 (0.96–0.99)	0.017	-	-
ALI	per 10 units	0.93 (0.88–0.98)	0.009	-	-	0.95 (0.91–1.00)	0.040	-	-	0.97 (0.91–1.02)	0.23	-	-

a Cox proportional hazards regression models were performed to evaluate the association between clinicopathological characteristics and survival outcomes. The outcomes are presented as hazard ratios (HRs) with 95% confidence intervals (CIs). Variables with a *P*-value of less than 0.10 in the univariate analysis were selected for inclusion in the multivariate regression models, using the backward conditional methods.

b All systemic nutrition/inflammation indicators were individually incorporated into the multivariate analyses.

AGR: albumin-to-globulin ratio; ALI: advanced lung cancer inflammation index; CONUT: controlling nutritional status score; COPD: chronic obstructive pulmonary disease; FEV1: forced expiratory volume in 1 s; FVC: forced vital capacity; GNRI: geriatric nutritional risk index; LMR: lymphocyte-to-monocyte ratio; NLR: neutrophil-to-lymphocyte ratio; NPR: neutrophil-to-platelet ratio; PLR: platelet-to-lymphocyte ratio; PNI: prognostic nutritional index; SII: systemic immune-inflammation index; SIRI: systemic inflammation response index.

**Figure 3:**
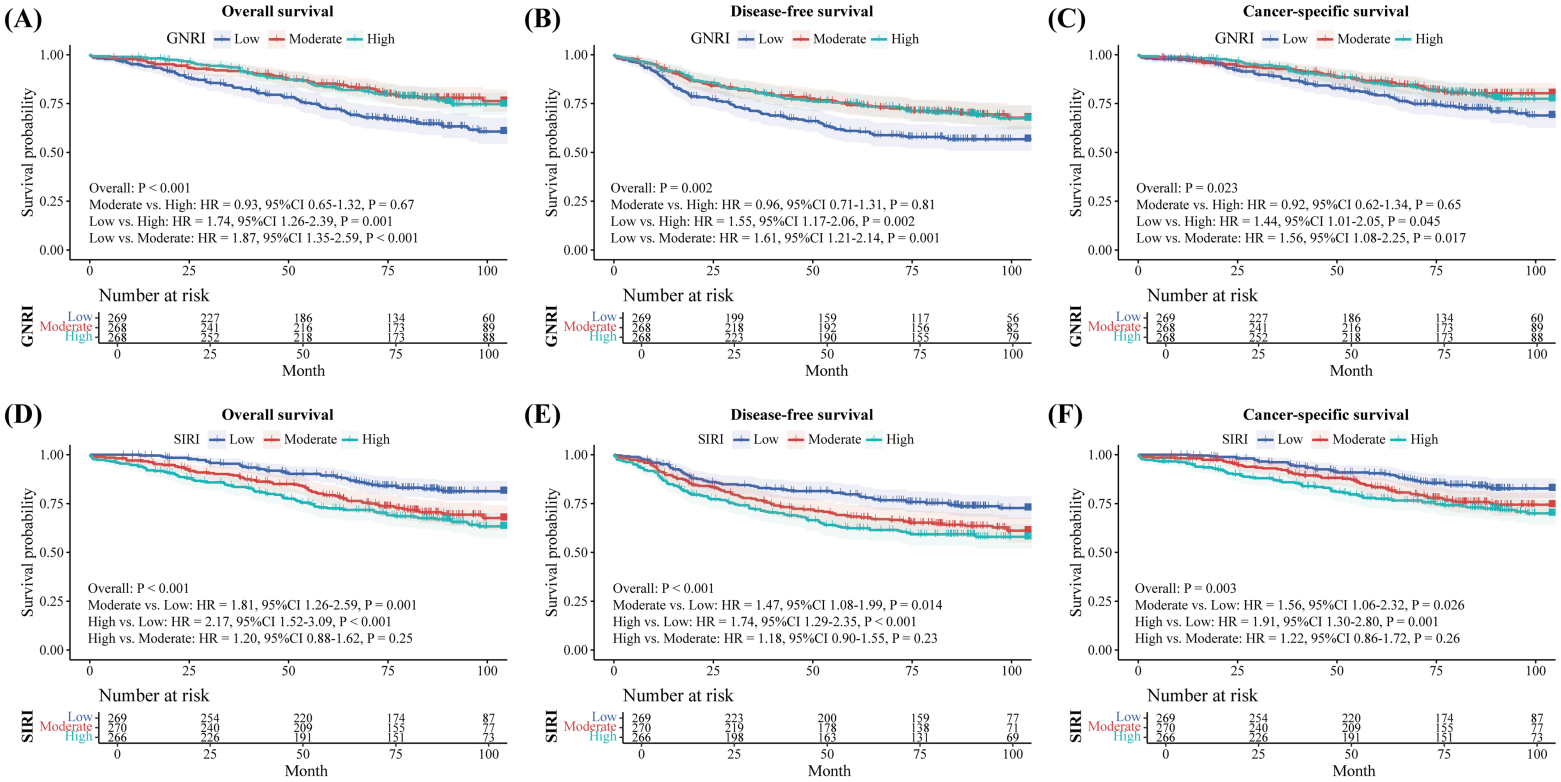
Survival analysis using the classification systems of GNRI and SIRI. Results of the Kaplan-Meier method with log-rank statistics are reported. The hazard ratios (HRs) with 95% confidence intervals (CIs) are derived from univariable Cox proportional hazards regression models. **A-C**: Analysis of overall survival **(A)**, disease-free survival **(B)**, and cancer-specific survival **(C)** using the GNRI classification system. **D-F**: Analysis of overall survival **(D)**, disease-free survival **(E)**, and cancer-specific survival **(F)** using the SIRI classification system. GNRI: geriatric nutritional risk index; SIRI: systemic inflammation response index.

The tertiles of GNRI and SIRI was used to classify the nutrition-inflammation status of NSCLC patients, respectively. The classification systems of GNRI and SIRI both demonstrated significant predictive values for OS, DFS, and CSS in NSCLC patients, but in different ways (Figure 3). A low GNRI and a high SIRI was associated with poor survival outcomes. Particularly, patients with a low GNRI had poorer OS, DFS, and CSS compared to those with a high or moderate GNRI, while no significant differences in these survival outcomes were observed between patients with a high or moderate GNRI.

### Changes in nutrition-inflammation status after surgery

The nutrition-inflammation status at 4 to 6 weeks following VATS lobectomy was recorded and assessed for 226 patients and compared to their preoperative status (Supplementary Table 3). No adjuvant therapy was administrated during the follow-up period after surgery. The nutritional indicators, including the levels of total proteins, serum proteins, and total lymphocytes, as well as the PNI and GNRI, significantly improved after surgical resection of the cancers. By contrast, the inflammatory indicators, including the levels of total neutrophils and total monocytes, as well as the NLR, LMR, and SIRI, remained stable during the observation period.

## Discussion

This study revealed that the 11 nutrition-inflammation indices possess moderate predictive value for both perioperative outcomes and long-term survival. The independent associations between these indices and therapeutic endpoints were generally weak or non-existent, suggesting that their applicability in clinical practice may be restricted.

Prior investigations have predominantly examined the influence of nutrition-inflammation status on survival in NSCLC patients individually, with limited comparative analyses across indices (14, 15). While extensive research has documented the association between systemic nutrition-inflammation profiles and survival outcomes, perioperative recovery has received less attention (1, 2). In NSCLC, elevated nutrition-inflammation risks have been linked to reduced treatment efficacy (14, 15), early recurrence (1), and poor survival rates (2). Similar adverse effects have been observed in other malignancies, including small cell lung cancer (24), gastrointestinal cancers (6, 22, 25), and urogenital cancers (26–28). For perioperative outcomes, high nutrition/inflammation risks have been identified as predictors of severe complications and infections in patients undergoing surgical resection for esophageal and gastric cancers (25, 29). This study compared 11 nutrition-inflammation indices for their ability to guide perioperative management and survival analysis. Although most indices showed associations with adverse outcomes, their predictive accuracy was generally low.

In our analysis, the GNRI exhibited the most optimal performance in predicting OS and DFS, while the LMR demonstrated the highest predictive capacity for postoperative complications and delayed hospital discharge. In contrast, Song et al. (2) compared the predictive values of 16 nutrition-inflammation indicators for OS in NSCLC patients and demonstrated the optimal performance of the ALI. Notably, the majority of the patients included in their study underwent first-line radiotherapy and/or chemotherapy, with only 22.7% undergoing surgery. This is significantly different from our study, which included solely surgically treated NSCLC patients. These differences may account for the different findings. However, both the study by Song et al. (2) and our study demonstrated a significant association between multiple nutrition-inflammation indices and OS, albeit with relatively low predictive accuracies. Additionally, Erciyestepe et al. (30) have preliminarily validated the association between the NLR, PLR, and PNI and survival and recurrence of NSCLC in a relatively small sample and without multivariable analysis. Regarding perioperative endpoints, Wang et al. (31) have highlighted the LMR for predicting postoperative complications by comparing different nutrition-inflammation indices in multicenter cohorts, which is consistent with our findings. The predictive accuracy of nutrition-inflammation indices for postoperative endpoints demonstrated by Wang et al. (31) was also relatively low. To address this, they introduced the parameter of serum total cholesterol to the LMR and established a new index called the “Systemic Nutrition-inflammation Index” to improve predictive performance (31). Their experience provides an example for improving and optimizing the existing nutrition-inflammation indices. From this perspective, our comparison of 11 nutrition-inflammation indices regarding both perioperative outcomes and survival endpoints in NSCLC patients provides benchmarks for future tool development. The combination of different nutrition-inflammation indices, as wells as the introduction of other nutrition-inflammation parameters or critical clinicopathological indicators, may help to enhance the predictive accuracy of therapeutic outcomes.

The predictive capabilities of these nutrition-inflammation indices are determined by their included parameters and calculation methods. Common parameters across these indices include serum albumin, total cholesterol, neutrophils, lymphocytes, monocytes, and body mass. Lymphocyte counts, comprising T cells, B cells, and NK cells, reflect immune system integrity (32, 33), while neutrophil and monocyte counts indicate inflammation levels (34). The LMR’s superior performance may stem from its ability to capture the interplay between lymphocytes, associated with immune surveillance and anti-tumor activity, and monocytes, linked to inflammation and tumor progression (35). A higher LMR suggests a more favorable immune status, potentially reducing postoperative complications and promoting recovery. The GNRI’s strong predictive performance for OS and DFS may be attributed to its comprehensive assessment of nutritional status through biochemical parameters (albumin) and anthropometric measures (body weight/ideal body weight) (8). Serum albumin is a well-established marker of nutritional status and immune function (36), while body weight/ideal body weight in the GNRI enhances its association with survival outcomes by reflecting body composition (29). This index is particularly relevant in NSCLC, where nutritional status is often compromised by chronic inflammation and tumor-related cachexia (37, 38). The GNRI’s ability to integrate nutritional and inflammatory aspects provides a holistic assessment of long-term prognosis. Overall, these indices reflect nutritional and immune properties, explaining their association with overall survival and weaker association with disease-free and cancer-specific survival.

Our study provides valuable insights into the potential application of nutrition-inflammation indices for perioperative management and prognosis assessment in NSCLC patients. However, the generally weak or absent independent associations between these indices and therapeutic endpoints highlight the need for caution in their application. The parameters included in these indices are primarily biochemical data, which are susceptible to influences from host characteristics such as age, gender, comorbidities, and cancer biology (1, 31). Based on the dynamic comparisons of nutrition-inflammation indicators (Supplementary Table 3), nutritional parameters have improved at 4 to 6 weeks after surgical resection of NSCLC, while inflammatory parameters remain stable. These fundings indicate that nutritional status can be significantly influenced by cancer biology and can quickly recover after cancer resection. By contrast, the inflammation status may depend more on patients’ intrinsic properties or may be persistently affected by cancer biology, such as minimal residual disease even after surgical resection (39), and warrants further investigation. These factors could partially explain the low predictive accuracy and the lack of independent association between nutrition-inflammation indices and therapeutic outcomes. Therefore, these indices should be used as part of a comprehensive assessment that incorporates clinical judgment and other relevant factors. Critical clinicopathological characteristics such as age, gender, and cancer stage, as identified in multivariable analysis (Tables 2, 3), may serve as valuable components in developing robust predictive models for therapeutic outcomes, which warranting systematic investigation. Future work should focus on enhancing the predictive value of nutrition-inflammation indices by selecting more relevant parameters or improving calculation and scoring systems.

### Limitations

Several limitations of this study warrant acknowledgment. Despite the relatively large sample size, the retrospective single-center design may introduce selection bias and limit the generalizability of the findings. The application of these nutrition-inflammation indices for assessing surgery risk and survival benefits requires further validation through prospective studies. In addition to the reported confounding factors, other potential influences, such as psychosocial status, cachexia, specific comorbidities, and oxygenation and capnography indicators, have not been comprehensively investigated. The dependence of nutrition-inflammation indicators on key clinicopathological factors, such as age, gender, smoking status, comorbidities, and cancer progression (1, 31), may account for the lack of independent predictive value and the modest predictive accuracy for therapeutic outcomes. On the other hand, the low AUC values observed may reflect the complex interplay between nutrition, inflammation, and tumor biology, which these indices cannot fully capture. Further research should explore more comprehensive biomarkers that integrate multiple aspects of the tumor microenvironment to improve the predictive accuracy. Moreover, combining multiple nutrition-inflammation indices or integrating them with critical clinicopathological parameters may improve perioperative management and prognosis analysis. In addition, the study focused on NSCLC patients undergoing VATS lobectomy, and the findings warranted validation in other surgical approaches or treatment modalities for NSCLC.

## Conclusion

This study demonstrates that nutrition-inflammation indices have moderate predictive value for perioperative endpoints and survival outcomes in NSCLC patients undergoing VATS lobectomy. The LMR is recommended for guiding perioperative management, while the GNRI is recommended for aiding in prognosis analysis. However, their utility in clinical practice may be limited due to the generally weak independent associations with therapeutic outcomes. Future studies should focus on validating these findings in prospective cohorts and exploring more comprehensive biomarkers to improve the accuracy of perioperative and prognostic assessments in NSCLC patients.

## Data Availability

The original contributions presented in the study are included in the article/Supplementary material, further inquiries can be directed to the corresponding authors.

## References

[1] Wang P Wang S Sun Z Li H Zhao Y Li Y. Systemic inflammation influences the prognosis of patients with radically resected non-small cell lung cancer and correlates with the immunosuppressive microenvironment. Int J Cancer. (2023) 153:826–42. doi: 10.1002/ijc.34547, PMID: 37186387

[2] Song M Zhang Q Song C Liu T Zhang X Ruan G. The advanced lung cancer inflammation index is the optimal inflammatory biomarker of overall survival in patients with lung cancer. J Cachexia Sarcopenia Muscle. (2022) 13:2504–14. doi: 10.1002/jcsm.13032, PMID: 35833264 PMC9530543

[3] Køstner AH Nielsen PS Georgsen JB Parner ET Nielsen MB Kersten C. Systemic inflammation associates with a myeloid inflamed tumor microenvironment in primary resected Colon Cancer—may cold tumors simply be too hot? Front Immunol. (2021) 12:716342. doi: 10.3389/fimmu.2021.716342, PMID: 34531864 PMC8438238

[4] Choi Y Kim JW Nam KH Han S-H Kim J-W Ahn S-H. Systemic inflammation is associated with the density of immune cells in the tumor microenvironment of gastric cancer. Gastric Cancer. (2016) 20:602–11. doi: 10.1007/s10120-016-0642-0, PMID: 27665104

[5] Li J Zhu N Wang C You L Guo W Yuan Z. Preoperative albumin-to-globulin ratio and prognostic nutritional index predict the prognosis of colorectal cancer: a retrospective study. Sci Rep. (2023) 13:17272. doi: 10.1038/s41598-023-43391-5, PMID: 37828259 PMC10570287

[6] Wang H-K Wei Q Yang Y-L Lu T-Y Yan Y Wang F. Clinical usefulness of the lymphocyte-to-monocyte ratio and aggregate index of systemic inflammation in patients with esophageal cancer: a retrospective cohort study. Cancer Cell Int. (2023) 23:13. doi: 10.1186/s12935-023-02856-3, PMID: 36707809 PMC9881346

[7] Liao G Zhao Z Yang H Chen M Li X. Can prognostic nutritional index be a prediction factor in esophageal Cancer?: a Meta-analysis. Nutr Cancer. (2019) 72:187–93. doi: 10.1080/01635581.2019.1631859, PMID: 31272238

[8] Bouillanne O Morineau G Dupont C Coulombel I Vincent JP Nicolis I. Geriatric nutritional risk index: a new index for evaluating at-risk elderly medical patients. Am J Clin Nutr. (2005) 82:777–83. doi: 10.1093/ajcn/82.4.777, PMID: 16210706

[9] Bray F Laversanne M Sung H Ferlay J Siegel RL Soerjomataram I. Global cancer statistics 2022: GLOBOCAN estimates of incidence and mortality worldwide for 36 cancers in 185 countries. CA Cancer J Clin. (2024) 74:229–263. doi: 10.3322/caac.21834, PMID: 38572751

[10] Pennathur A Brunelli A Criner GJ Keshavarz H Mazzone P Walsh G. Definition and assessment of high risk in patients considered for lobectomy for stage I non–small cell lung cancer: the American Association for Thoracic Surgery expert panel consensus document. J Thorac Cardiovasc Surg. (2021) 162:1605–18.e6. doi: 10.1016/j.jtcvs.2021.07.030, PMID: 34716030

[11] Chen K Wang X Yang F Li J Jiang G Liu J. Propensity-matched comparison of video-assisted thoracoscopic with thoracotomy lobectomy for locally advanced non-small cell lung cancer. J Thorac Cardiovasc Surg. (2017) 153:967–976.e2. doi: 10.1016/j.jtcvs.2016.12.008, PMID: 28088426

[12] Suzuki K Saji H Aokage K Watanabe SI Okada M Mizusawa J. Comparison of pulmonary segmentectomy and lobectomy: safety results of a randomized trial. J Thorac Cardiovasc Surg. (2019) 158:895–907. doi: 10.1016/j.jtcvs.2019.03.090, PMID: 31078312

[13] Wood SL Pernemalm M Crosbie PA Whetton AD. The role of the tumor-microenvironment in lung cancer-metastasis and its relationship to potential therapeutic targets. Cancer Treat Rev. (2014) 40:558–66. doi: 10.1016/j.ctrv.2013.10.001, PMID: 24176790

[14] Yang H Wang K Li B Li S Li Y Yuan L. The prognostic role of blood inflammatory biomarkers and EGFR mutation status in stage IIIA/N2 non-small cell lung Cancer patients treated with Trimodality therapy. Front Oncol. (2021) 11:707041. doi: 10.3389/fonc.2021.707041, PMID: 34917497 PMC8668866

[15] Mountzios G Samantas E Senghas K Zervas E Krisam J Samitas K. Association of the advanced lung cancer inflammation index (ALI) with immune checkpoint inhibitor efficacy in patients with advanced non-small-cell lung cancer. ESMO Open. (2021) 6:100254. doi: 10.1016/j.esmoop.2021.100254, PMID: 34481329 PMC8417333

[16] von Elm E Altman DG Egger M Pocock SJ Gøtzsche PC Vandenbroucke JP. The strengthening the reporting of observational studies in epidemiology (STROBE) statement: guidelines for reporting observational studies. Lancet. (2007) 370:1453–7. doi: 10.1016/S0140-6736(07)61602-X 18064739

[17] Harimoto N Yoshizumi T Inokuchi S Itoh S Adachi E Ikeda Y. Prognostic significance of preoperative controlling nutritional status (CONUT) score in patients undergoing hepatic resection for hepatocellular carcinoma: a multi-institutional study. Ann Surg Oncol. (2018) 25:3316–23. doi: 10.1245/s10434-018-6672-6, PMID: 30051372

[18] Ma SJ Yu H Khan M Gill J Santhosh S Chatterjee U. Evaluation of optimal threshold of neutrophil-lymphocyte ratio and its association with survival outcomes among patients with head and neck Cancer. JAMA Netw Open. (2022) 5:e227567. doi: 10.1001/jamanetworkopen.2022.7567, PMID: 35426920 PMC9012962

[19] Zhang Y Peng W Zheng X. The prognostic value of the combined neutrophil-to-lymphocyte ratio (NLR) and neutrophil-to-platelet ratio (NPR) in sepsis. Sci Rep. (2024) 14:15075. doi: 10.1038/s41598-024-64469-8, PMID: 38956445 PMC11219835

[20] Diem S Schmid S Krapf M Flatz L Born D Jochum W. Neutrophil-to-lymphocyte ratio (NLR) and platelet-to-lymphocyte ratio (PLR) as prognostic markers in patients with non-small cell lung cancer (NSCLC) treated with nivolumab. Lung Cancer. (2017) 111:176–81. doi: 10.1016/j.lungcan.2017.07.024, PMID: 28838390

[21] Hu B Yang XR Xu Y Sun YF Sun C Guo W. Systemic immune-inflammation index predicts prognosis of patients after curative resection for hepatocellular carcinoma. Clin Cancer Res. (2014) 20:6212–22. doi: 10.1158/1078-0432.CCR-14-0442, PMID: 25271081

[22] Qi Q Zhuang L Shen Y Geng Y Yu S Chen H. A novel systemic inflammation response index (SIRI) for predicting the survival of patients with pancreatic cancer after chemotherapy. Cancer. (2016) 122:2158–67. doi: 10.1002/cncr.30057, PMID: 27152949

[23] Gilbert A Piccinin C Velikova G Groenvold M Kuliś D Blazeby JM. Linking the European Organisation for Research and Treatment of Cancer item library to the common terminology criteria for adverse events. J Clin Oncol Off J Am Soc Clin Oncol. (2022) 40:3770–80. doi: 10.1200/JCO.21.02017, PMID: 35973158 PMC9649281

[24] Qi WX Xiang Y Zhao S Chen J. Assessment of systematic inflammatory and nutritional indexes in extensive-stage small-cell lung cancer treated with first-line chemotherapy and atezolizumab. Cancer Immunol Immunother. (2021) 70:3199–206. doi: 10.1007/s00262-021-02926-3, PMID: 33796915 PMC10991671

[25] Okugawa Y Toiyama Y Yamamoto A Shigemori T Ichikawa T Yin C. Lymphocyte-to-C-reactive protein ratio and score are clinically feasible nutrition-inflammation markers of outcome in patients with gastric cancer. Clin Nutr. (2020) 39:1209–17. doi: 10.1016/j.clnu.2019.05.009, PMID: 31155370

[26] Xu T Zhang S-M Wu H-M Wen X-M Qiu D-Q Yang Y-Y. Prognostic significance of prognostic nutritional index and systemic immune-inflammation index in patients after curative breast cancer resection: a retrospective cohort study. BMC Cancer. (2022) 22:1128. doi: 10.1186/s12885-022-10218-x, PMID: 36329394 PMC9632068

[27] Nie D Gong H Mao X Li Z. Systemic immune-inflammation index predicts prognosis in patients with epithelial ovarian cancer: a retrospective study. Gynecol Oncol. (2019) 152:259–64. doi: 10.1016/j.ygyno.2018.11.034, PMID: 30558974

[28] Khan AI Psutka SP Patil DH Hong G Williams MA Bilen MA. Sarcopenia and systemic inflammation are associated with decreased survival after cytoreductive nephrectomy for metastatic renal cell carcinoma. Cancer. (2022) 128:2073–84. doi: 10.1002/cncr.34174, PMID: 35285950

[29] Wang PY Chen XK Liu Q Xu L Zhang RX Liu XB. Application of four nutritional risk indexes in perioperative management for esophageal cancer patients. J Cancer Res Clin Oncol. (2021) 147:3099–111. doi: 10.1007/s00432-021-03585-8, PMID: 33687565 PMC7941130

[30] Erciyestepe M Selvi O Dinç Sonuşen Ş Öztürk AE Dinç G Güneş TK. Prognostic value of inflammation and nutrition-based scores in non-small cell lung Cancer. Med Princ Pract. (2024) 33:122–32. doi: 10.1159/000535781, PMID: 38091965 PMC11095608

[31] Wang P Wang S Huang Q Chen X Yu Y Zhang R. Development and validation of the systemic nutrition/inflammation index for improving perioperative management of non-small cell lung cancer. BMC Med. (2025) 23:113. doi: 10.1186/s12916-025-03925-2, PMID: 39988705 PMC11849302

[32] Pearce EL Poffenberger MC Chang CH Jones RG. Fueling immunity: insights into metabolism and lymphocyte function. Science. (2013) 342:1242454. doi: 10.1126/science.1242454, PMID: 24115444 PMC4486656

[33] Gruver AL Hudson LL Sempowski GD. Immunosenescence of ageing. J Pathol. (2007) 211:144–56. doi: 10.1002/path.2104, PMID: 17200946 PMC1931833

[34] Templeton AJ McNamara MG Seruga B Vera-Badillo FE Aneja P Ocana A. Prognostic role of neutrophil-to-lymphocyte ratio in solid tumors: a systematic review and meta-analysis. J Natl Cancer Inst. (2014) 106:dju124. doi: 10.1093/jnci/dju124, PMID: 24875653

[35] Tan D Fu Y Tong W Li F. Prognostic significance of lymphocyte to monocyte ratio in colorectal cancer: a meta-analysis. Int J Surg. (2018) 55:128–38. doi: 10.1016/j.ijsu.2018.05.030, PMID: 29807167

[36] Hirata T Arai Y Yuasa S Abe Y Takayama M Sasaki T. Associations of cardiovascular biomarkers and plasma albumin with exceptional survival to the highest ages. Nat Commun. (2020) 11:3820. doi: 10.1038/s41467-020-17636-0, PMID: 32732919 PMC7393489

[37] Al-Sawaf O Weiss J Skrzypski M Lam JM Karasaki T Zambrana F. Body composition and lung cancer-associated cachexia in TRACERx. Nat Med. (2023) 29:846–58. doi: 10.1038/s41591-023-02232-8, PMID: 37045997 PMC7614477

[38] Yang M Shen Y Tan L Li W. Prognostic value of sarcopenia in lung cancer: a systematic review and meta-analysis. Chest. (2019) 156:101–11. doi: 10.1016/j.chest.2019.04.115, PMID: 31128115

[39] Pellini B Chaudhuri AA. Circulating tumor DNA minimal residual disease detection of non-small-cell lung Cancer treated with curative intent. J Clin Oncol. (2022) 40:567–75. doi: 10.1200/JCO.21.01929, PMID: 34985936 PMC8853615

