# Supplementary material for: Comparing 11 nutrition-inflammation indices for perioperative management and prognostic evaluation in non-small cell lung cancer patients
Source: Front Nutr. 2025 Jun 4;12:1577563. doi: 10.3389/fnut.2025.1577563 (PMC12173913; doi:10.3389/fnut.2025.1577563)
Supplement: Supplementary file 1 [file Table_1.docx]

**Supplementary Table 1. Calculation of 11 systemic nutrition/inflammation indicators.**

| Systemic nutrition/inflammation indicators | Abbreviations | Calculation methods |
| --- | --- | --- |
| Prognostic nutritional index | PNI | 10 × serum albumin (g/dL) + 0.005 × total lymphocytes |
| Controlling nutritional status score | COUNT | Calculated based on levels of serum albumin, total lymphocytes, and total cholesterol |
| Albumin-to-globulin ratio | AGR | Serum albumin / serum globulin |
| Neutrophil-to-lymphocyte ratio | NLR | Total neutrophils / total lymphocytes |
| Platelet-to-lymphocyte ratio | PLR | Total platelets / total lymphocytes |
| Neutrophil-to-platelet ratio | NPR | Total neutrophils / total platelets |
| Lymphocyte-to-monocyte ratio | LMR | Total lymphocytes / total monocytes |
| Systemic inflammation response index | SIRI | Total neutrophils × total monocytes / total lymphocytes |
| Systemic immune-inflammation index | SII | Total neutrophils × total platelets / total lymphocytes |
| Geriatric nutritional risk index | GNRI | 1.489 × serum albumin (g/dL) + 41.7 × (present weight/ideal weight) |
| Advanced lung cancer inflammation index | ALI | Body mass index (kg/m^2^) ×serum albumin (g/dL) / (total neutrophils/total lymphocytes) |
