# Supplementary material for: Comparing 11 nutrition-inflammation indices for perioperative management and prognostic evaluation in non-small cell lung cancer patients
Source: Front Nutr. 2025 Jun 4;12:1577563. doi: 10.3389/fnut.2025.1577563 (PMC12173913; doi:10.3389/fnut.2025.1577563)
Supplement: Supplementary file 2 [file Table_2.docx]

**Supplementary Table 2: Predictive values of systemic nutrition-inflammation indicators for perioperative and survival outcomes after VATS lobectomy in NSCLC patients (N = 805).**

| Parameters | Incidence of overall complications^1^ | |  | Delayed hospital discharge^1^ | |  | Overall survival^2^ | | |  | Disease-free survival^2^ | | |  | Cancer-specific survival^2^ | | |
| --- | --- | --- | --- | --- | --- | --- | --- | --- | --- | --- | --- | --- | --- | --- | --- | --- | --- |
|  | AUC (95% CI) | *P* values |  | AUC (95% CI) | *P* values |  | 3-year | 5-year | 8-year |  | 3-year | 5-year | 8-year |  | 3-year | 5-year | 8-year |
| ***Biochemical groups*** |  |  |  |  |  |  |  |  |  |  |  |  |  |  |  |  |  |
| PNI | 0.568 (0.517-0.619) | 0.009 |  | 0.566 (0.525-0.607) | 0.002 |  | 0.612 | 0.585 | 0.599 |  | 0.552 | 0.572 | 0.567 |  | 0.585 | 0.554 | 0.566 |
| COUNT | 0.590 (0.539-0.641) | 0.001 |  | 0.565 (0.524-0.607) | 0.002 |  | 0.559 | 0.555 | 0.526 |  | 0.528 | 0.531 | 0.513 |  | 0.542 | 0.535 | 0.500 |
| AGR | 0.504 (0.451-0.557) | 0.87 |  | 0.510 (0.467-0.553) | 0.63 |  | 0.624 | 0.568 | 0.600 |  | 0.569 | 0.558 | 0.572 |  | 0.597 | 0.558 | 0.587 |
| NLR | 0.569 (0.520-0.619) | 0.008 |  | 0.562 (0.521-0.603) | 0.004 |  | 0.622 | 0.600 | 0.560 |  | 0.567 | 0.574 | 0.536 |  | 0.622 | 0.585 | 0.530 |
| PLR | 0.543 (0.493-0.592) | 0.10 |  | 0.547 (0.506-0.588) | 0.027 |  | 0.515 | 0.533 | 0.518 |  | 0.511 | 0.525 | 0.511 |  | 0.504 | 0.518 | 0.510 |
| NPR | 0.549 (0.500-0.598) | 0.058 |  | 0.523 (0.482-0.565) | 0.48 |  | 0.620 | 0.594 | 0.549 |  | 0.564 | 0.570 | 0.531 |  | 0.632 | 0.589 | 0.542 |
| LMR | 0.625 (0.579-0.671) | <0.001 |  | 0.606 (0.566-0.645) | <0.001 |  | 0.625 | 0.618 | 0.592 |  | 0.558 | 0.588 | 0.566 |  | 0.625 | 0.604 | 0.567 |
| SIRI | 0.591 (0.542-0.640) | <0.001 |  | 0.575 (0.534-0.615) | <0.001 |  | 0.640 | 0.629 | 0.585 |  | 0.580 | 0.596 | 0.556 |  | 0.653 | 0.617 | 0.555 |
| SII | 0.534 (0.483-0.585) | 0.19 |  | 0.540 (0.499-0.582) | 0.060 |  | 0.593 | 0.587 | 0.539 |  | 0.563 | 0.568 | 0.520 |  | 0.586 | 0.572 | 0.510 |
| ***Biochemical and anthropometry groups*** | | |  |  |  |  |  |  |  |  |  |  |  |  |  |  |  |
| GNRI | 0.571 (0.519-0.622) | 0.007 |  | 0.546 (0.503-0.588) | 0.033 |  | 0.658 | 0.631 | 0.584 |  | 0.585 | 0.600 | 0.563 |  | 0.653 | 0.610 | 0.550 |
| ALI | 0.589 (0.540-0.638) | 0.001 |  | 0.573 (0.532-0.614) | 0.001 |  | 0.643 | 0.599 | 0.584 |  | 0.574 | 0.590 | 0.576 |  | 0.607 | 0.571 | 0.548 |

^1^ Receiver Operating Characteristic (ROC) curves were utilized to evaluate the predictive values of nutrition-inflammation indicators for the incidence of postoperative complications and delayed hospital discharge.

^2^ Time-dependent ROC curves were employed to assess the predictive value of nutrition-inflammation indicators concerning the survival outcomes. Area under the curve (AUC) values are reported for these analyses.

AGR: albumin-to-globulin ratio; ALI: advanced lung cancer inflammation index; AUC: area under the curve; CI: confidence interval; CONUT: controlling nutritional status score; GNRI: geriatric nutritional risk index; LMR: lymphocyte-to-monocyte ratio; NLR: neutrophil-to-lymphocyte ratio; NPR: neutrophil-to-platelet ratio; PLR: platelet-to-lymphocyte ratio; PNI: prognostic nutritional index; SII: systemic immune-inflammation index; SIRI: systemic inflammation response index.
