# Supplementary material for: Comparing 11 nutrition-inflammation indices for perioperative management and prognostic evaluation in non-small cell lung cancer patients
Source: Front Nutr. 2025 Jun 4;12:1577563. doi: 10.3389/fnut.2025.1577563 (PMC12173913; doi:10.3389/fnut.2025.1577563)
Supplement: Supplementary file 3 [file Table_3.docx]

**Supplementary Table 3. Postoperative changes of systemic nutrition/inflammation indicators in NSCLC patients undergoing VATS lobectomy (*N* = 226).**

| Indicators | Before surgery | After surgery | *P* values |
| --- | --- | --- | --- |
| ***Nutrition/inflammation items*** | | | |
| Total protein, g/L | 65.8 (63.4-71.1) | 70.7 (66.9-74.4) | <0.001 |
| Serum albumin, g/L | 41.3 (38.9-44.0) | 43.6 (41.5-45.6) | <0.001 |
| Serum globulin, g/L | 25.1 (22.7-28.7) | 26.8 (23.9-29.9) | <0.001 |
| Total neutrophils, /mm^3^ | 3690 (2700-4730) | 3690 (3008-4560) | 0.12 |
| Total lymphocytes, /mm^3^ | 1580 (1280-1990) | 1710 (1278-2163) | 0.014 |
| Total monocytes, /mm^3^ | 475 (340-530) | 480 (348-553) | 0.66 |
| ***Representative nutrition-inflammation indexes*** | | | |
| PNI | 49.4 (46.0-52.9) | 52.2 (49.4-55.1) | <0.001 |
| AGR | 1.63 (1.48-1.78) | 1.63 (1.44-1.84) | 0.82 |
| NLR | 2.27 (1.62-3.26) | 2.25 (1.62-3.06) | 0.92 |
| LMR | 3.60 (2.63-4.93) | 3.57 (2.98-5.00) | 0.47 |
| SIRI | 0.979 (0.656-1.538) | 0.986 (0.685-1.341) | 0.80 |
| GNRI | 107 (101-114) | 111 (104-115) | <0.001 |
| ALI | 44.4 (28.9-63.3) | 48.1 (34.4-66.4) | 0.43 |

Data are shown as median (interquartile range). P values are from paired sample Wilcoxon signed-rank tests (P).

AGR: albumin-to-globulin ratio; ALI: advanced lung cancer inflammation index; GNRI: geriatric nutritional risk index; LMR: lymphocyte-to-monocyte ratio; NLR: neutrophil-to-lymphocyte ratio; PNI: prognostic nutritional index; SIRI: systemic inflammation response index.
